# The Neuroprotective Effects of Exosomes Derived from TSG101-Overexpressing Human Neural Stem Cells in a Stroke Model

**DOI:** 10.3390/ijms23179532

**Published:** 2022-08-23

**Authors:** Eun-Jung Yoon, Yunseo Choi, Tae Myoung Kim, Ehn-Kyoung Choi, Yun-Bae Kim, Dongsun Park

**Affiliations:** 1 Department of Biology Education, Korea National University of Education, Cheongju 28173, Korea; 2 Department of Counseling, Health, and Kinesiology, College of Education and Human Development, Texas A&M University-San Antonio, One University Way, San Antonio, TX 78224, USA; 3 Central Research Institute, Designed Cells Co., Ltd., Cheongju 28576, Korea; 4 College of Veterinary Medicine, Chungbuk National University, Cheongju 28644, Korea

**Keywords:** exosome, TSG101, neural stem cells, MCAO, neuroprotection

## Abstract

Although tissue-type plasminogen activator was approved by the FDA for early reperfusion of occluded vessels, there is a need for an effective neuroprotective drug for stroke patients. In this study, we established tumor susceptibility gene (TSG)101-overexpressing human neural stem cells (F3.TSG) and investigated whether they showed enhanced secretion of exosomes and whether treatment with exosomes during reperfusion alleviated ischemia-reperfusion-mediated brain damage. F3.TSG cells secreted higher amounts of exosomes than the parental F3 cells. In N2A cells subjected to oxygen–glucose deprivation (OGD), treatment with exosomes or coculture with F3.TSG cells significantly attenuated lactate dehydrogenase release, the mRNA expression of proinflammatory factors, and the protein expression of DNA-damage-related proteins. In a middle cerebral artery occlusion (MCAO) rat model, treatment with exosomes, F3 cells, or F3.TSG cells after 2 h of occlusion followed by reperfusion reduced the infarction volume and suppressed inflammatory cytokines, DNA-damage-related proteins, and glial fibrillary acidic protein, and upregulated several neurotrophic factors. Thus, TSG101-overexpressing neural stem cells showed enhanced exosome secretion; exosome treatment protected against MCAO-induced brain damage via anti-inflammatory activities, DNA damage pathway inhibition, and growth/trophic factor induction. Therefore, exosomes and F3.TSG cells can affect neuroprotection and functional recovery in acute stroke patients.

## 1. Introduction

Stroke is a commonly occurring fatal disease and a leading cause of disability worldwide [1]. Clinically, ischemic stroke accounts for approximately 80% of all strokes, and results from occlusion of the major cerebral artery by a thrombus or emboli [2]. Currently, the therapeutic strategy for acute ischemic stroke is recanalization or thrombolytic therapy by intravenous injection of thrombolytic agents [3]. Tissue plasminogen activator (t-PA) is a thrombolytic agent and the only licensed therapy approved by the Food and Drug Administration (FDA) for this condition [4]. However, the optimal time for treatment with t-PA is 4.5 h after the onset of stroke; fast reperfusion contributes to secondary injury including neuronal death and extensive infarction induced by inflammatory cytokines, dysregulation of the DNA damage pathway, and excessive generation of reactive oxygen species (ROS) [5,6,7,8,9].

Recently, exosomes have received considerable attention owing to their biological functions. Exosomes are a subspecies of extracellular vesicles, approximately 30–150 nm in diameter, and are released into the extracellular fluids by cells in all living systems [10]. They contain cytokines, growth factors, signaling lipids, and genetic materials, and play essential roles in intercellular communication by transferring their cargo between source and target cells under physiological and pathophysiological conditions [11]. Accumulating evidence has shown that treatment with exosomes exerts anti-inflammatory effects and contributes to DNA damage repair and cell proliferation. Several studies have reported that exosomes regulate intercellular communication among components of the neurovascular unit after stroke. For example, exosomes derived from mesenchymal stem cells attenuated inflammation by transferring miR-34a, miR-124, and miR-135b [12]. The miR-1246 in exosomes regulates DNA damage by targeting DNA ligase 4 (LIG4) [13]. Exosomes isolated from hypoxia-preconditioned mesenchymal stem cells rescued synaptic dysfunction and promoted anti-inflammatory effects in an animal model of Alzheimer’s disease [14].

Several studies have been conducted on the production and isolation of exosomes. Stem cells are stimulated to secrete exosomes when grown as three-dimensional cultures [15]. In addition, hypoxia enhances the production of exosomes by regulating hypoxia-inducible factor (HIF) [16]. Multiple mechanisms involved in exosome biogenesis have been identified. The endosomal sorting complex required for transport (ESCRT) machinery plays a prominent role in this process, and the soluble N-ethylmaleimide-sensitive factor attachment protein receptor (SNARE) proteins and their effectors such as Ras-related in brain (Rab) GTPases participate prominently in exosomal secretion [17,18]. The tumor susceptibility gene 101 (TSG101) and signal-transducing adaptor molecules play critical roles in exosome biogenesis and secretion [19]. For example, the downregulation of TSG101 reduces exosome secretion in tumor and dendritic cells [19,20]. Additionally, leptin enhances exosome release by increasing TSG101 expression [21]. 

A previous study confirmed that the overexpression of functional genes in neural stem cells enhanced their biological function. Neural stem cells transfected with choline acetyltransferase improved cognitive function in an Alzheimer’s disease animal model by increasing the concentration of acetylcholine [22]. In addition, neural stem cells upregulated Olig2 and improved neurobehavioral function in an experimental model of neonatal periventricular leukomalacia [23]. Therefore, in the present study, we investigated whether neural stem cells overexpressing TSG101 can show upregulated exosome biogenesis and exhibit neuroprotective effects. We successfully established a TSG101-overexpressing human neural stem cell line that shows upregulated exosome production. Our results show that these cells and the exosomes secreted by these cells exhibit neuroprotective effects in vitro in an N2A neuroblastoma cell model as well as in vivo in a rat middle cerebral artery occlusion (MCAO) model. The data also reveal that the underlying mechanism of action involves the modulation of inflammatory markers and neurotrophic factors both at the mRNA and protein levels.

## 2. Results

### 2.1. Establishment of F3.TSG Cells

**Figure 1:**
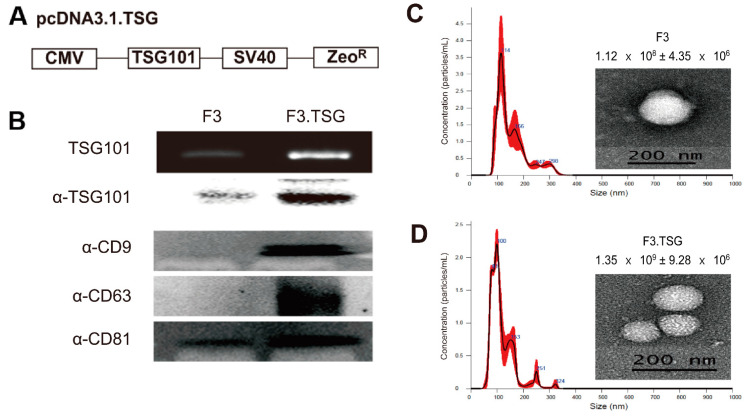
Establishment of F3.TSG. (**A**) Construct of pcDNA3.1_TSG. (**B**) Expression of exosome marker (TSG101, CD9, CD63 and CD81) in F3 and F3.TSG by RT-PCR and western blotting. (**C**,**D**) Number of exosomes by nanoparticle tracking analysis in F3 (**C**) and F3.TSG cells (**D**). All samples were analyzed in triplicate.

As shown Figure 1A, the construct of a pcDNA3.1_TSG vector was designed. An RT–PCR analysis confirmed the stable overexpression of the TSG101 gene in F3 human NSCs (Figure 1B); the expression of TSG101 mRNA in F3.TSG cells is depicted in Figure 1B. A Western blotting also demonstrated a strong expression of the TSG101 protein in F3.TSG cells (Figure 1B).

In next-generation RNA-Seq profiling, a heat map analysis revealed that 202 genes were associated with the overexpression of TSG101, and 159 genes were upregulated (Supplementary Figure S1A). Among them, *CALR, CAPN2, DNM2, ENO1, SFN, PDIA3, HSPA1A, HSPD1, RPS3*, and *TPT1* were found to be related to the cell death pathway as per the biological process analysis (Supplementary Figure S1B). The cellular components associated with exosome biogenesis are shown in Supplementary Figure S1C. Additionally, a molecular function analysis revealed that they were binding proteins (Supplementary Figure S1D). The interactive graph showed results consistent with those obtained from the GO analysis (Supplementary Figure S1E). These results suggest that the overexpression of TSG101 enhanced the secretion of exosomes. 

To confirm the enhancement of the secretion of exosomes derived from F3.TSG cells, an electron microscope and nanoparticle tracking analysis was performed (Figure 1C,D). For each cell type (F3 and F3.TSG), we harvested exosomes from media 3 days after the culture attained 90–100% confluence. The secretion of exosomes from F3.TSG cells was approximately 10-fold higher than that from F3 cells. Moreover, other exosome markers such as CD9, CD63, and CD81 were increased in F3.TSG compared with F3 cells.

### 2.2. Neuroprotective and Anti-Inflammatory Effects of Exosomes in OGD-Exposed N2A Cells

**Figure 2:**
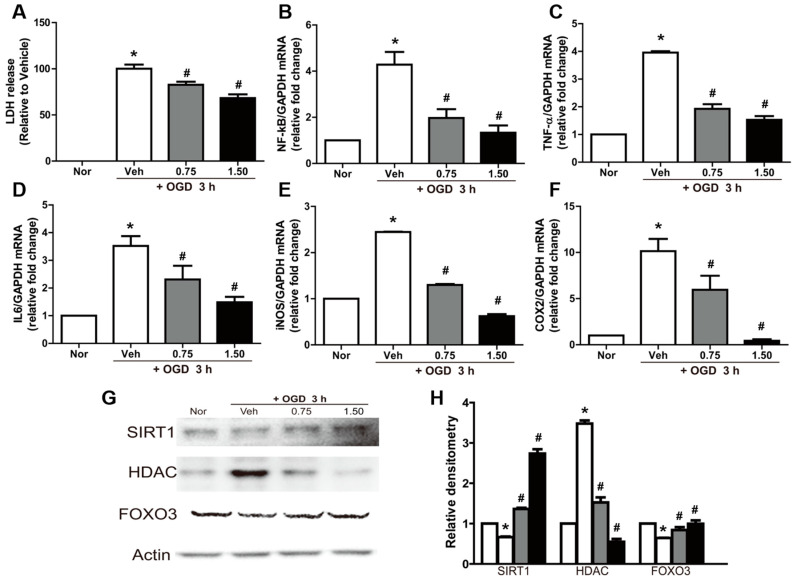
Neuroprotective and anti-inflammatory effects of exosomes in N2A cells subjected to oxygen–glucose deprivation (OGD). (**A**) Neuroprotective effects of exosomes under 3 h OGD exposure in the lactate dehydrogenase (LDH) assay; (**B**–**F**) real-time PCR analysis of mRNA of nuclear factor (NF)-κB (**B**), tumor necrosis factor (TNF)-α (**C**), interleukin (IL)-6 (**D**), inducible nitric oxide synthase (iNOS) (**E**), and cyclooxygenase 2 (COX2) (**F**) normalized to glyceraldehyde-3-phosphate dehydrogenase (GAPDH); (**G**,**H**) Western blot analysis of sirutin 1 (SIRT1), histone deacetylase (HDAC), and forkhead box O-3 (FOXO3) showing representative bands (**G**) and band densities normalized to actin (**H**). Densitometric analysis of the Western blot was performed using ImageJ. n = 3 per treatment group. * Significantly different from normal control (*p* < 0.05). # Significantly different from vehicle control (*p* < 0.05).

Exposure of N2A cells to OGD significantly increased LDH release (Figure 2A). The cytotoxicity induced by OGD was significantly attenuated by exosome treatment in a dose-dependent manner. 

In addition, exosome treatment led to anti-inflammatory effects. The exposure of N2A cells to OGD conditions significantly upregulated NF-κB expression (Figure 2B). Interestingly, NF-κB expression was markedly attenuated by exosome treatment. In parallel with the change in NF-κB, the expression of TNF-α and IL-6 was also remarkably increased by OGD, which was blocked by treatment with exosomes (Figure 2C,D). The gene expression of the inflammatory enzymes iNOS and COX2 was also increased following exposure to OGD; OGD regulated the expression of proinflammatory proteins such as iNOS and COX2 (Figure 2E,F). Treatment with exosomes significantly inhibited the expression of these enzymes in a concentration-dependent manner.

A Western blot analysis revealed that the production of proteins related to DNA damage such as sirutin 1 (SIRT1) and forkhead box O-3 (FOXO3) was decreased by OGD, while HDAC expression was upregulated (Figure 2G,H). However, the OGD-induced abnormal production of the above proteins was markedly attenuated by treatment with exosomes, although there were differences in sensitivity.

### 2.3. Reversal of OGD-Induced Effects by Exosomes in N2A Cells

**Figure 3:**
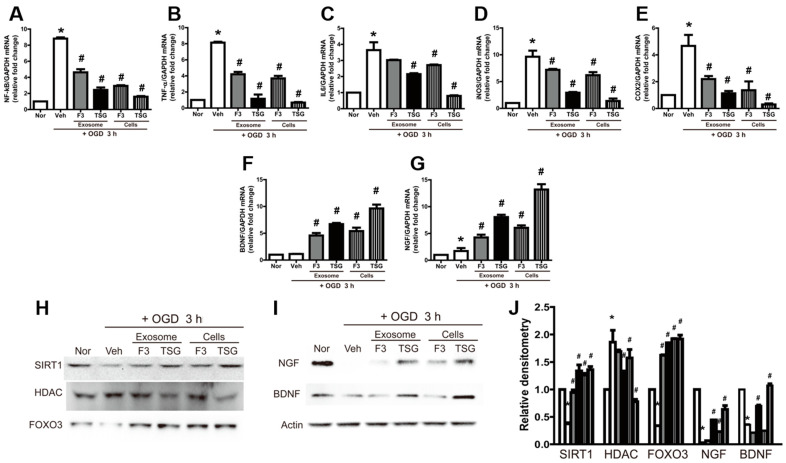
Reversal of OGD-induced changes by exosomes in N2A cells. (**A**–**G**) Real-time PCR analysis of mRNA of the inflammatory factors nuclear factor (NF)-κB (**A**), tumor necrosis factor (TNF)-α (**B**), interleukin 6 (IL-6) (**C**), inducible nitric oxide synthase (iNOS) (**D**), and cyclooxygenase 2 (COX2) (**E**) and the neurotrophin family members brain-derived neurotrophic factor (BDNF) (**F**) and nerve growth factor (NGF) (**G**). They were normalized to glyceraldehyde-3-phosphate dehydrogenase (GAPDH); (**H**–**J**) Western blot analysis of sirtuin 1 (SIRT1), histone deacetylase (HDAC), and forkhead box O-3 (FOXO3) proteins showing representative bands (**H**), NGF and BDNF showing representative bands (**I**); band densities normalized to actin (**J**). Densitometric analysis of the Western blot was performed using ImageJ. n = 3 per treatment group. * Significantly different from normal control (*p* < 0.05). # Significantly different from vehicle control (*p* < 0.05).

To investigate whether exosomes were able to reverse the effects of 3 h of OGD, OGD-exposed N2A cells subjected to OGD were cocultured with F3 and F3.TSG on 0.4 μM and 4.0 μM membrane filters for 24 h with fresh media in 5% CO2 in an incubator at 37 °C. Data showed that the expression of markers of inflammation such as NF-κB, TNF-α, IL-6, iNOS, and COX2 increased following exposure to OGD conditions and reoxygenation (Figure 3A–E). However, the coculture of N2A cells with F3 and F3.TSG led to a decrease in their expression; F3.TSG cells were superior to the F3 parental cells in this regard. Additionally, coculture on the 4.0 μM membrane filter had a more potent effect than that on the 0.4 μM membrane filter. The expression of BDNF and NGF was also remarkably increased by coculture with F3 and F3.TSG cells (Figure 3F,G). 

A Western blot analysis showed that SIRT1 and FOXO3 levels were decreased and HDAC levels were increased by OGD and reoxygenation (Figure 3H). However, the OGD-induced abnormal production of the proteins was markedly attenuated by coculture with F3 and F3.TSG cells, although there were differences in sensitivity. In addition, BDNF and NGF protein levels were decreased by OGD and reoxygenation, and this downregulation of the proteins was markedly reversed by coculture with F3 and F3.TSG cells (Figure 3I).

### 2.4. Neuroprotective Effects of Exosomes in MCAO Animals

**Figure 4:**
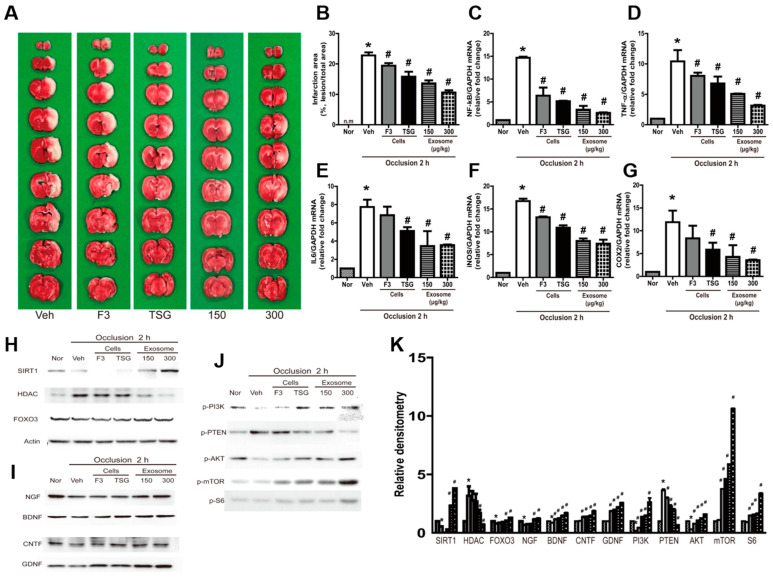
Neuroprotective effects of exosomes in middle cerebral artery occlusion (MCAO) animals. (**A**) Brain infarction volume in stroke-challenged rats treated with F3 cells, F3.TSG cells, or exosomes (0.5 or 1.0 mL/kg). Representative images of brain sections stained with 2% 2,3,5-triphenyltetrazolium chloride; (**B**) total infarction volumes were summed up from all slices; (**C**–**G**) real-time PCR analysis of mRNA of nuclear factor (NF)-κB (**C**), tumor necrosis factor α (TNF-α) (**D**), interleukin 6 (IL-6) (**E**), inducible nitric oxide synthase (iNOS) (**F**), and cyclooxygenase 2 (COX2) (**G**) normalized to glyceraldehyde-3-phosphate dehydrogenase (GAPDH); (**H**–**K**) Western blot analysis of the following growth/trophic factor proteins: sirtuin 1 (SIRT1), histone deacetylase (HDAC), and forkhead box O-3 (FOXO3) for DNA damage (**H**), nerve growth factor (NGF), brain-derived neurotrophic factor (BDNF), ciliary neurotrophic factor (CNTF), and glial-cell-derived neurotrophic factor (GDNF) (**I**); representative bands of the following proliferation-related proteins phosphatidyl inositol-3-phosphate kinase (PI3K), phosphatase and tensin homolog (PTEN), protein kinase B (AKT), mammalian target of rapamycin (mTOR), and S6 (**J**); the band densities normalized to actin (**K**). n = 3 per treatment group. * Significantly different from normal control (*p* < 0.05). # Significantly different from vehicle control (*p* < 0.05).

Brain injury was evaluated by quantifying the total infarction volume in brain tissue. The typical TTC (red) staining patterns of the brain tissues are illustrated in Figure 4A. The pale infarction volume in MCAO rats was approximately 22.8% of the total brain volume (Figure 4B). However, treatment with F3 or F3.TSG cells during reperfusion following 2 h of ischemia markedly decreased the infarction volume to 19.4% and 15.8%, respectively. Additionally, treatment with 150 or 300 μg/kg exosomes derived from F3.TSG cells significantly decreased the infarction volume to 13.6% and 10.6%, respectively. 

The expression of genes related to inflammation such as NF-κB, TNF-α, IL-6, iNOS, and COX2 was markedly upregulated in MCAO brains (Figure 4C–G). This upregulation was significantly inhibited by treatment with F3 cells, F3.TSG cells, or exosomes. F3.TSG cells were superior to F3 cells in this regard. Treatment with 1.0 mL/kg exosomes induced the most potent neuroprotective effect.

A Western blot analysis showed that SIRT1 and FOXO3 were decreased in MCAO brains while HDAC was increased (Figure 4H). However, alterations in these proteins were normalized by treatment with F3 cells, F3.TSG cells, or exosomes. In addition, growth and neurotrophic factors, including NGF, BDNF, CNTF, and GDNF, were increased by treatment with F3 or F3.TSG cells, or exosomes (Figure 4I). Proteins in the cell proliferation signaling pathway including phosphatidyl inositol-3-phosphate kinase (PI3K), phosphatase and tensin homolog (PTEN), protein kinase B (AKT), mammalian target of rapamycin (mTOR), and S6 were altered by treatment with F3 cells, F3.TSG cells, or exosomes (Figure 4J).

## 3. Discussion

The MCAO model shows biochemical and pathological features similar to those observed in human stroke, including oxidative stress and inflammatory responses. In the present study, it was demonstrated that the overexpression of TSG101 in neural stem cells enhanced the secretion of exosomes, and treatment with exosomes during reperfusion alleviated ischemia-reperfusion-mediated brain damage in rats. The concentration of exosomes were measured by size, particle number, and exosome markers [24]. Especially, in the coculture with TSG101 cells, since exosomes are smaller than 0.4 μm, only exosomes were secreted at the 0.4 μm membrane, and at the 4.0 μm membrane, cells and exosomes affected N2A cells together [25,26]. These results were consistent with those obtained in N2A cells subjected to OGD and in the brain after MCAO.

TSG101 is a highly conserved protein in mice and humans, and is a component of the ESCRT machinery [27]. Although it was first reported as a virus-binding protein [28], it has recently been shown that TSG101 plays important roles in recognizing and sorting ESCRT cargo [29]. The biogenesis and secretion of exosomes is mainly regulated by the ESCRT machinery. In the ESCRT pathway, phosphatidylinositol-3-phosphate (PI3P) promotes cargo formation through interaction with hepatocyte receptor tyrosine kinase substrate (HRS) [30]. ESCRT-0 recruits ESCRT-I by interacting with TSG101. ESCRT-I and -II promote endosomal inward budding around clusters of ubiquitinated proteins. Subsequently, ESCRT-III binds to ESCRT-II. Finally, the bud is cleaved to form exosomes by the addition of charged multivesicular body protein 3 (CHAMP3), followed by ESCRT-III disassembly using ATP, catalyzed by vacuolar sorting protein 4 (VPS4) [30]. There is much evidence to support the critical role of ESCRT in exosome biogenesis. For example, the loss of HRS, signal transducing adapter molecule 1 (STAM1), and TSG101 reduces exosome secretion in multiple cell types such as tumor cells and dendritic cells [19]. Leptin, a hormone that regulates energy, balance, and hunger, enhances exosome release by increasing TSG101 expression [20]. Indeed, TSG101 is used as an exosome biomarker, and in sequencing results, the downregulation of calreticulin (CALR) has been shown to influence protein secretion through the exosome pathway [31]. Calpain 2 (CAPN2) and protein disulfide-isomerase A3 (PDIA3) have been identified in exosomes derived from breast cancer cells [32]. The upregulation of dynamin 2 (DNM2) correlates with increased exosome uptake [33]. Sulforaphane (SFN) induces exosome-mediated paracrine senescence during cancer therapy [34]. The localization of heat shock protein family A (Hsp70) member 1A (HSPA1A) is related to exosomes [35]. Heat shock protein family D (Hsp60) member 1 (HSPD1) has been observed on the surface of exosomes [36]. Ribosomal protein S3 (RPS3) is a highly expressed protein in the exosomes of gastric cancer cells [37]. Tumor protein, translationally controlled 1 (TPT1) is exported via exosomes from blood outgrowth endothelial cells [38]. Therefore, the overexpression of TSG101 in neural stem cells enhances the gene related with exosome biogenesis and then enhances the secretion of exosomes. 

Many studies are being conducted to explore the application of exosomes in cancer treatment. Exosomes are rich in small RNA molecules such as miRNAs and mRNAs [39]. They also contain cytokines such as IL-1 and growth/tropic factors, such as those from the neurotrophin family [40]. Several studies have confirmed that treatment with exosomes attenuates inflammation and brain damage induced by MCAO. miR-98 reduces neuronal damage in ischemic stroke by inhibiting the inflammatory pathway [41]. miR-146a-5p reduces microglial-mediated neuroinflammation after ischemic stroke [42]. The levels of proinflammatory mediators in serum exosomes are associated with worsened stroke outcomes in C3aR-dependent microglial phagoptosis [43]. The delivery of NGF mRNA and protein via exosomes facilitates the recovery from brain damage by cerebral ischemia [44]. In this study, exosomes derived from F3.TSG cells showed potent neuroprotective effects following MCAO. Generally, cytokines such as TNF-α and IL-6 are upregulated in the brain after a stroke [45]. Proinflammatory cytokines such as TNF-α exacerbate cerebral damage, whereas anti-inflammatory cytokines may be neuroprotective, suggesting that inflammatory responses during ischemia and reperfusion are important processes in neural injury [46]. Indeed, a previous study demonstrated that the intravenous infusion of NSCs during reperfusion reduced microglial activation, decreased the expression of proinflammatory factors, and ameliorated damage to the blood–brain barrier [7]. Similarly, in the present study, treatment with exosomes reversed the upregulation of TNF-α and IL-6. In addition, exosome treatment led to the downregulation of the expression of inflammatory mediators such as NF-κB, iNOS, and COX2. The increased expression of GFAP in activated astrocytes is a marker of neuroinflammation and secondary brain injury [47]. This astrocytic activation was effectively inhibited by F3.TSG cell and exosome treatment. 

Sirt1, a member of the HDAC superfamily, plays an important role in neuroprotection and is related to anti-inflammatory effects in cerebral ischemia because its inhibition exacerbates ischemic injury accompanied by an increased acetylation of NF-κB [48]. In addition, Sirt1 increases the activity of DNA repair pathways such as the poly [ADP-ribose] polymerase 1 (PARP1) pathway associated with stroke [9]. Therefore, Sirt1 is considered to be a therapeutic target for ischemic stroke. Moreover, HDAC1 participates in the DNA damage repair pathway and cell survival [49]. Its inhibition leads to anti-inflammatory and neuroprotective effects in stroke [48]. Foxo3 upregulation protects neurons against hypoxia-induced brain injury [50]. In this study, treatment with exosomes also affected the Sirt1/HDAC pathway and protected against MCAO-induced brain damage. In addition, TSG101 expression was able to ameliorate brain damage because TSG101 is associated with HDAC activity. 

Neurotrophins play a key role in the protection and recovery of neurons after stroke [51]. BDNF is responsible for the development, differentiation, maintenance, and repair of neurons [52]. It has also been reported that intravenous injection of BDNF reduces infarcts in temporary focal cerebral ischemia [53]. NGF is well known for its neuroprotective functions following ischemic brain damage. NGF reduces inflammation by reshaping microglial polarization and promoting cell survival after cerebral ischemia [44]. Additionally, intracerebral infusion of GDNF promotes striatal neurogenesis after stroke [54] and CNTF has been shown to promote the development of neurons and glial cells and prevent ischemia-reduced neuronal loss in gerbils [55,56]. In our previous study, vascular endothelial growth factor (VEGF), GDNF, NGF, and CNTF were enhanced by the infusion of F3.ChAT cells during reperfusion and exerted neuroprotective effects [7]. In the present study, we demonstrated that treatment with exosomes showed remarkable neuroprotective effects through the production of large amounts of NGF, BDNF, GDNF, and CNTF.

To date, exosomes have relied on mass production by mass culture. Although, we need more study before applying for a clinical trial, we demonstrated that the upregulation of TSG101 enhances the secretion of exosomes. Furthermore, these results suggest that exosome-derived stem cells protects against ischemia-induced brain damage via anti-inflammatory effects and the production of growth/trophic factors. Therefore, exosomes derived from F3.TSG and F3.TSG cells are good candidates for developing treatments for acute ischemic stroke patients.

## 4. Materials and Methods

### 4.1. TSG101 Plasmid Construction

For the overexpression of TSG101 (NM_006292), cDNA clones of human TSG101 including BamHI (N-terminal) and XbaI (C-terminal) sites were obtained from Bioneer Corp. (Daejeon, Korea). The cDNA inserts were cloned into the appropriate pcDNA3.1 plasmid vector (Invitrogen, Carlsbad, CA, USA) using the BamHI and XbaI cloning sites. The recombinant plasmids were transformed into DH5α bacteria (Real Biotech Corporation, Banqiao City, Taiwan), and the bacteria were grown in Luria–Bertani (LB) broth containing 50 μg/mL ampicillin at 37 °C. After inoculation with a single colony, plasmid DNA was extracted using a miniprep kit (Real Biotech Corporation) according to the manufacturer’s protocol. After sequence confirmation, the plasmid DNA was extracted using a midiprep kit (Qiagen, Hilden, Germany) according to the manufacturer’s protocol.

### 4.2. Establishment of the F3.TSG Cell Line

The establishment of the F3 neural stem cell line has been described previously [57]. For the stable overexpression of TSG101, F3 cells were plated in 6-well plates before transfection with the recombinant plasmid DNA using Lipofectamine2000 (Invitrogen). Twenty-four hours after transfection, the cells were continuously cultured in a medium containing 2.0 μg/mL zeocin (Invitrogen). The surviving cells were cultured into cell lines stably expressing TSG101. To confirm stable expression, cells were analyzed by RT-PCR and Western blotting. The cells confirmed to stably express TGS101 were defined as F3.TSG cells.

### 4.3. Cell Culture

N2A (mouse neuroblastoma cell line, ATCC, Manassas, VA, USA), F3 (human neural stem cell), and F3.TSG (TSG101-overexepressing F3 cells) cells were cultured in Dulbecco’s modified Eagle’s medium (DMEM; Biowest, Nuaillé, Cholet, France) containing antibiotics (100 IU/mL penicillin and 100 μg/mL streptomycin) and 10% heat-inactivated fetal bovine serum (Biowest) at 37 °C in a 5% CO_2_/95% air atmosphere. In all experiments, cells were grown until more than 90% confluence and subjected to no more than 20 passages.

### 4.4. Exosome Isolation

F3 and F3.TSG cells were seeded in Hyperflasks (Corning, NY, USA) with 2.5 × 10^7^ cells and cultured for 2 days in an incubator until 90–100% confluence. To obtain conditioned medium for exosome isolation, the cultures were washed 3 times using 300 mL PBS each time, and the medium was replaced with 500 mL of phenol red-free DMEM; the cells were subsequently cultured for 3 days in an incubator. Then, the conditioned media were filtered using a 0.2 μm filter top system (Corning) to remove cell debris [58]. Then, the filtered media were subjected to ultracentrifugation (120,000× *g* at 4 °C for 2 h) to collect exosome. The total exosomes were concentrated 10 times (*v*/*v*) using Vivaflow 200 (M.W. cutoff 100 kDa, Sartorius AG, Gottingen, Germany), and freeze-dried (LP-10; Ilshinbiobase Co., Ltd., Seoul, South Korea). The isolated exosomes were stored at −80 °C and resuspended in PBS for treatment. 

### 4.5. Nanoparticle Tracking Analysis

Exosomes in the freeze-dried medium were analyzed using a NanoSight NS 300 instrument (NanoSight Ltd., Amesbury, UK). The analysis settings were optimized and kept constant between samples, and the mean, mode, median, and estimated concentration for each particle size were determined. As per published methods [59], all analyses were carried out with samples at a 1:1000 dilution, yielding particle concentrations in the region of 1 × 10^8^ particles mL^−1^ (in accordance with the manufacturer’s recommendations). All samples were analyzed in triplicate. 

### 4.6. Transcriptome Sequencing Analysis

RNA samples from F3 and F3.TSG cells were sent to Macrogen Inc. (Seoul, Korea) for transcriptome sequencing and generation of datasets. A TruSeq Stranded mRNA LT Sample Prep Kit (Illumina, San Diego, CA, USA) was used to prepare a cDNA library according to the TruSeq Stranded mRNA Sample Preparation Guide (Part # 15031047 Rev. E). Briefly, DNase was used to remove DNA contamination. The poly-A mRNAs were then purified, and the mRNA was broken down into short fragments. The cleaved RNA fragments were used to synthesize cDNA using random hexamer primers. After purification, the cDNA fragments were ligated to universal adapters containing sequencing priming sites. Subsequently, the fragments were amplified by PCR and analyzed by agarose gel electrophoresis. Fragments with insert sizes between 200 and 400 bp were subjected to deep sequencing. For paired-end sequencing, both ends of the cDNA were sequenced using an Illumina NovaSeq 6000 instrument (Illumina, San Diego, CA, USA). For quality control of the sequenced raw reads, the overall read quality, total bases, total reads, GC (%), and basic statistics were calculated. To reduce biases in the analysis, artifacts such as low-quality reads, adaptor sequences, contaminant DNA, and PCR duplicates were removed using the Trimmomatic program [60]. The sliding window method was used, and bases of reads that did not qualify within a window size of 4 and mean quality of 15 were trimmed [60]. Reads with lengths shorter than 36 bp were dropped to produce trimmed data. The trimmed reads were aligned using the HISAT2 version 2.1.0 software (Johns Hopkins University, Baltimore, MD, USA). FASTQ files were mapped using Homo Sapiens software. Hg38 was used as the reference library, and gene annotations were added to the read count values using NCBI_109.20200522. Subsequently, the number of reads that mapped to each gene was counted using StringTie version 2.1.3b (Johns Hopkins University, Baltimore, MD, USA); this software tool measures the abundance of each transcript as fragments per kilobase of transcript per million mapped reads (FPKM). 

Genes whose expression displayed an average fold change >2 that was statistically significant (adjusted *p* value ≤ 0.05) were defined as differentially expressed (DEGs). To better understand the biological functions and metabolic pathways of the identified genes, the DEGs were functionally classified using a Gene Ontology (GO) analysis using ShinyGO (http://bioinformatics.sdstate.edu/go/ (accessed on 1 February 2022)) and GOnet (https://tools.dice-database.org/GOnet/ (accessed on 1 February 2022)).

### 4.7. Oxygen–Glucose Deprivation (OGD) Culture of N2A Cells and Treatment with Exosomes

Glucose-free phenol red-free DMEM (Sigma-Aldrich, St. Louis, MO, USA) was employed as the OGD medium. Before hypoxia culture, the culture media were flushed with nitrogen gas for 30 min. The cells were then subjected to hypoxia by placing them for 3 h in a chamber filled with 8% O_2_ and 92% N_2_. 

To investigate the neuroprotective effect of exosomes, N2A cells were seeded in 10 cm dishes. After a 24 h incubation, the cells were washed twice with OGD media and treated with 0, 0.75, and 1.50 (μg/mL) of exosomes for 3 h under OGD conditions. The concentration of exosome was measured by the BCA method. Then, the media were used to analyze lactate dehydrogenase (LDH) concentration, and the cells were used for performing real-time PCR and Western blotting.

To investigate the effect of exosomes and F3.TSG cells on OGD-exposed N2A cells, N2A cells were seeded in 6-well plates. After a 24 h incubation period, the cells were washed twice with OGD medium and incubated for 3 h under OGD conditions. The cells were then washed twice with PBS and replaced with fresh normal DMEM. The N2A cells were then cocultured with F3 and F3.TSG cells. The F3 and F3.TSG cells were seeded in the upper chambers of 6-well transwell plates (pore size 0.4 and 4.0 μm). After a 24 h incubation period, the N2A cells were subjected to real-time PCR and Western blotting analyses.

### 4.8. Lactate Dehydrogenase (LDH) Assay in N2A Cells

The protective effect of exosomes was quantified by measuring LDH release from N2A cells. LDH content was determined using a commercial nonradioactive LDH assay kit (Promega, Madison, WI, USA); this method is based on a coupled enzymatic reaction that converts a tetrazolium salt into a red formazan product. The increase in the amount of formazan produced in the culture supernatant directly correlates with an increase in the number of lysed cells. The formazan was quantified spectrophotometrically by measuring the absorbance at 490 nm (Molecular Devices, Sunnyvale, CA, USA). The experiments were performed in triplicate.

### 4.9. Animals

Eight-week-old male Sprague-Dawley rats (n = 10/group) weighing 300–330 g were purchased from Daehan Biolink (Eumseong, Korea). Rats were housed in an environmentally controlled room with a constant temperature (23 ± 3 °C) and relative humidity (50 ± 10%), and a 12 h light/dark cycle. They were fed a standard rodent diet and purified water ad libitum. All animal experiments were performed in accordance with the Standard Operation Procedures of the Laboratory Animal Center, Chungbuk National University (CBNU), Korea. The protocol was approved by the Institutional Animal Care and Use Committee of CBNU (#CBNUA-1527-21-02).

### 4.10. Treatment of the Middle Cerebral Artery Occlusion Model (MCAO) with Exosomes and Cells 

Silicone-coated threads were prepared according to a previously described method [61]. Briefly, polyethylene tubing (Intramedic, Batavia, IL, USA) with an internal diameter of 0.28 mm was filled with silicone. A 4/0 monofilament nylon suture (Ailee, Busan, Korea) was inserted 5 mm into one end of the polyethylene tubing [62]. After insertion, the tube and encased silicone were cut using a razor blade at a point 0.5 mm beyond the tip of the nylon suture, and the silicone was dried for 24 h. Immediately prior to surgery, the polyethylene tube was removed from the nylon suture, leaving a uniform coating of silicone bonded to the distal 5 mm.

Focal cerebral ischemia was induced in rats as described previously [63] with slight modifications [7,8,62]. Briefly, rats were anesthetized with isoflurane and restrained in the supine position. A midline incision was made on the ventral side of the neck, exposing the left common carotid artery (CCA), external carotid artery (ECA), and internal carotid artery (ICA). The ICA and the lower part of the CCA were blocked using a clip, and the upper part of the ECA was ligated. Subsequently, an opening was made in the middle of the ECA, and a silicone-coated thread was introduced through the opening. The thread was advanced 18 mm via the ICA up to the origin of the MCA. After occlusion was achieved, the thread was secured in place using a ligature, and the incision was sutured. The silicone thread was removed for reperfusion after 2 h occlusion.

F3 and F3.TSG cells were suspended in an appropriate volume of saline (1 × 10^6^ cells/100 μL/rat) for infusion. The cells and exosomes (150 and 300 μg/kg) were intravenously infused into the animals via the jugular vein following reperfusion.

### 4.11. 2,3,5-Triphenyltetrazolium Chloride (TTC) Staining

Twenty-four hours after MCAO surgery, the rats were sacrificed under deep anesthesia with diethyl ether. The brains were carefully dissected into 2 mm coronal sections using a stainless-steel matrix. Each section was placed in a 24-well plate and incubated in 2% TTC solution for 15 min at 37 °C [7,8,62]. Gentle stirring of the plates ensured even exposure to the stain. After staining, excess TTC solution was drained, and the slices were fixed in 10% neutral buffered formalin. The infarction area in each section was determined using ImageJ software (version 1.53, National Institutes of Health, Bethesda, MD, USA). The total infarct volume was calculated by summing the infarction areas in each section and multiplying by the slice thickness (2 mm).

### 4.12. Quantitative Real-Time PCR Analysis of N2A Cells and Brain Tissues

Total RNA was isolated from N2A cells and brain tissues using TRIzol Reagent (Invitrogen) according to the manufacturer’s instructions. Quantitative real-time PCR was performed as previously described [64]. Glyceraldehyde 3-phosphate dehydrogenase (GAPDH) was used as an internal standard to normalize the expression of the target transcripts. Primer sets were used to amplify nuclear factor-κB (NF-κB), tumor necrosis factor (TNF)-α, interleukin (IL)-6, inducible nitric oxide synthase (iNOS), and cyclooxygenase 2 (COX2) (Supplementary Table S1). Triplicate data from five independent assays were analyzed using a comparative Ct method [64].

### 4.13. Western Blot Analysis of N2A Cells and Brain Tissues

N2A cells and brain tissues were homogenized in 10 volumes of radioimmunoprecipitation assay (RIPA) buffer (Thermo Scientific, Waltham, MA, USA) containing protease inhibitors (Sigma-Aldrich, St. Louis, MO, USA) and phosphatase inhibitors (Sigma-Aldrich, St. Louis, MO, USA). A Western blot analysis was performed as previously described [64]. The membranes were immunoblotted with primary antibodies, followed by incubation with horseradish peroxidase-conjugated antirabbit and antimouse secondary antibodies (Jackson ImmunoResearch Laboratories, Inc., West Grove, PA, USA). The antibodies used in this study are listed in Supplementary Table S2. The band densities were measured using ImageJ software (version 1.53, National Institutes of Health, Bethesda, MD, USA) and normalized to the density of actin.

### 4.14. Statistical Analysis

Statistical comparisons between groups were performed using a one-way analysis of variance (ANOVA) followed by Tukey’s multiple comparison test. All analyses were conducted using Statistical Package for Social Sciences for Windows software (version 12.0; SPSS Inc., Chicago, IL, USA). Statistical significance was set at *p* < 0.05. All data are expressed as the mean ± SD.

## 5. Conclusions

In this study, we demonstrated that the upregulation of TSG101 enhanced the secretion of exosomes and infusion of F3.TSG cells protects against MCAO-induced brain damage during reperfusion via anti-inflammatory effects and the production of growth/trophic factors. Therefore, exosomes derived from F3.TSG and F3.TSG cells are good candidates for developing treatments for acute ischemic stroke patients.

## Data Availability

Data are contained within the article or Supplementary Materials.

## Supplementary Materials

The supporting information can be downloaded at: https://www.mdpi.com/article/10.3390/ijms23179532/s1.
