# Supplementary material for: The Neuroprotective Effects of Exosomes Derived from TSG101-Overexpressing Human Neural Stem Cells in a Stroke Model
Source: Int J Mol Sci. 2022 Aug 23;23(17):9532. doi: 10.3390/ijms23179532 (PMC9455780; doi:10.3390/ijms23179532)
Supplement: Supplementary file 1 [file ijms-23-09532-s001.zip › ijms-1881018-supplementary.pdf]

Supplementary Table S1. Sequences of the primers used in the current study.

Gene Name	Accession No.	Human Primer	
		Forward (5′-3′)	Reverse (5′-3′)
TSG101	NM_006292	TTCTCAGCCTCCTGTGACCACT	CCATTTCCTCCTTCATCCGCCA
GAPDH	NM_001289746	AAGAAGGTGGTGAAGCAG	GTCAAAGGTGGAGGAGTG
Gene Name	Accession No.	Mouse Primer	
		Forward (5′-3′)	Reverse (5′-3′)
NF-κB	NM_008689	CACTGCTCAGGTCCACTGTC	CTGTCACTATCCCGGAGTTCA
TNF-α	NM_013693	TACCTTGTTGCCTCCTCTT	GTCACCAAATCAGCGTTATTAAG
IL-6	NM_031168.1	TCCAGTTGCCTTCTTGGGAC	AGTCTCCTCTCCGGACTTGT
iNOS	NM_010927.3	CTATGGCCGCTTTGATGTGC	TTGGGATGCTCCATGGTCAC
COX2	NM_011198	GAACCTGCAGTTTGCTGTGG	ACTCTGTTGTGCTCCCGAAG
GAPDH	NM_008084	CGTGCCGCCTGGAGAAACC	TGGAAGAGTGGGAGTTGCTGTTG
Gene Name	Accession No.	Rat Primer	
		Forward (5′-3′)	Reverse (5′-3′)
NF-κB	NM_001276711	AGAGGCCATTGAAGTGATCCA	CTTGTGGAGGAGGACGAGAGA
TNF-α	NM_012675	ATCGGTCCCAACAAGGAGGA	TTGCTACGACGTGGGCTAC
IL-6	NM_012589	ACCCCAACTTCCAATGCTCTC	ATGGTCTTGGTCCTTAGCCAC
iNOS	NM_012611	AGGCTGGAAGCCGTAACAAA	ACCACTGAATCCTGCCGATG
COX2	NM_017232	TGATCTACCCTCCCCACGTC	CACTCTGTTGTGCTCCCGAA
GAPDH	NM_017008	GTCGGTGTGAACGGATTTGG	CCACTTTGTCACAAGAGAAGGCA

Supplementary Table S2. List of antibodies used in the current study.

Epitope	Company	Cat. Number	Dilution	2° Ab (IgG)
TSG101 (44 kDa)	abcam	ab83	1:1000	anti-mouse
SIRT1 (81 kDa)	abcam	ab156585	1:5000	anti-rabbit
HDAC (60 kDa)	abcam	ab53091	1:500	anti-rabbit
FOXO3 (71 kDa)	abcam	ab154786	1:5000	anti-rabbit
NGF (13 kDa)	abcam	ab6199	1:1000	anti-rabbit
BDNF (28 kDa)	abcam	ab226843	1:1000	anti-rabbit
CNTF (24 kDa)	abcam	ab270992	1:1000	anti-rabbit
GDNF (24 kDa)	abcam	ab176564	1:5000	anti-rabbit
PI3K (85 kDa)	Cell Signaling Technology	#4228	1:1000	anti-rabbit
PTEN (54 kDa)	Cell Signaling Technology	#9554	1:1000	anti-rabbit
AKT (60 kDa)	Cell Signaling Technology	#9271	1:1000	anti-rabbit
mTOR (289 kDa)	Cell Signaling Technology	#2971	1:1000	anti-rabbit
S6 (32 kDa)	Cell Signaling Technology	#2211	1:1000	anti-rabbit
Actin (42 kDa)	Cell Signaling Technology	#5125	1:5000	HRP-conjugation

A

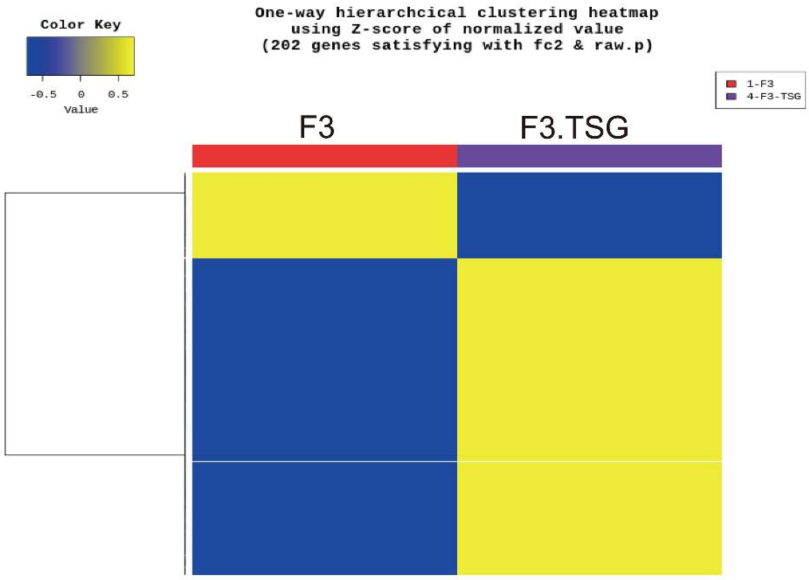

Color Key
-0.5 0 0.5
Value
One-way hierarchcical clustering heatmap
using Z-score of normalized value
(202 genes satisfying with fc2 & raw.p)
1-F3
4-F3-TSG
F3
F3.TSG

B

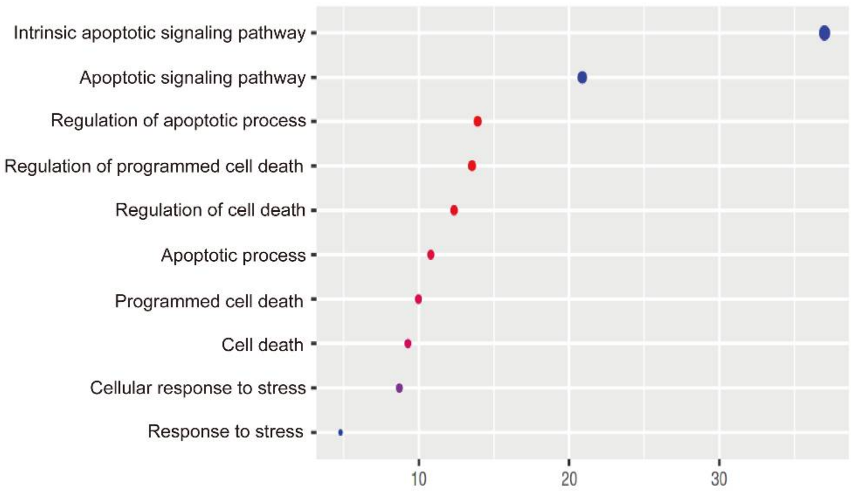

Intrinsic apoptotic signaling pathway
Apoptotic signaling pathway
Regulation of apoptotic process
Regulation of programmed cell death
Regulation of cell death
Apoptotic process
Programmed cell death
Cell death
Cellular response to stress
Response to stress
10
20
30

C

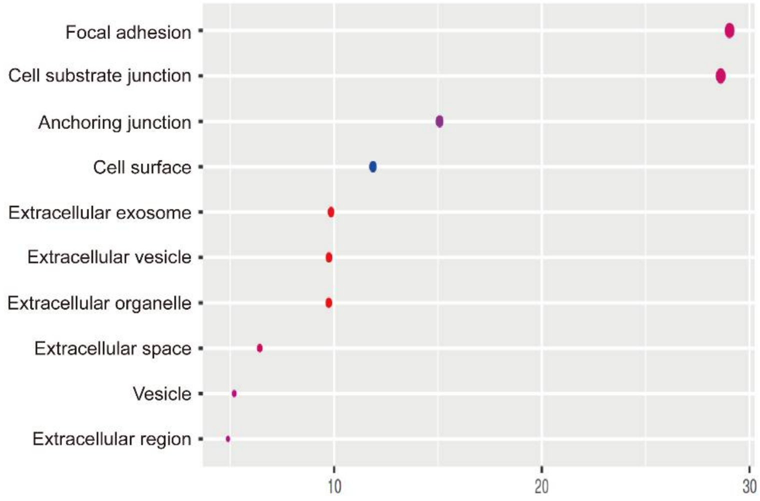
Focal adhesion
Cell substrate junction
Anchoring junction
Cell surface
Extracellular exosome
Extracellular vesicle
Extracellular organelle
Extracellular space
Vesicle
Extracellular region
10
20
30

D

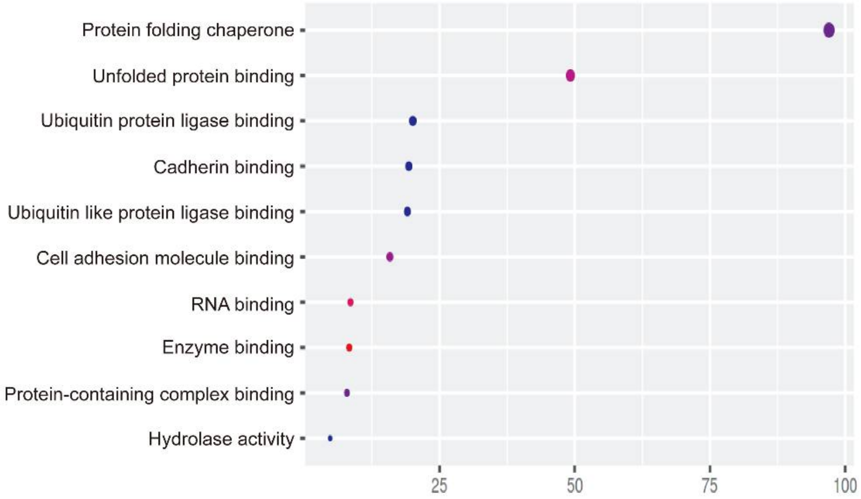
Protein folding chaperone
Unfolded protein binding
Ubiquitin protein ligase binding
Cadherin binding
Ubiquitin like protein ligase binding
Cell adhesion molecule binding
RNA binding
Enzyme binding
Protein-containing complex binding
Hydrolase activity
25
50
75
100

E

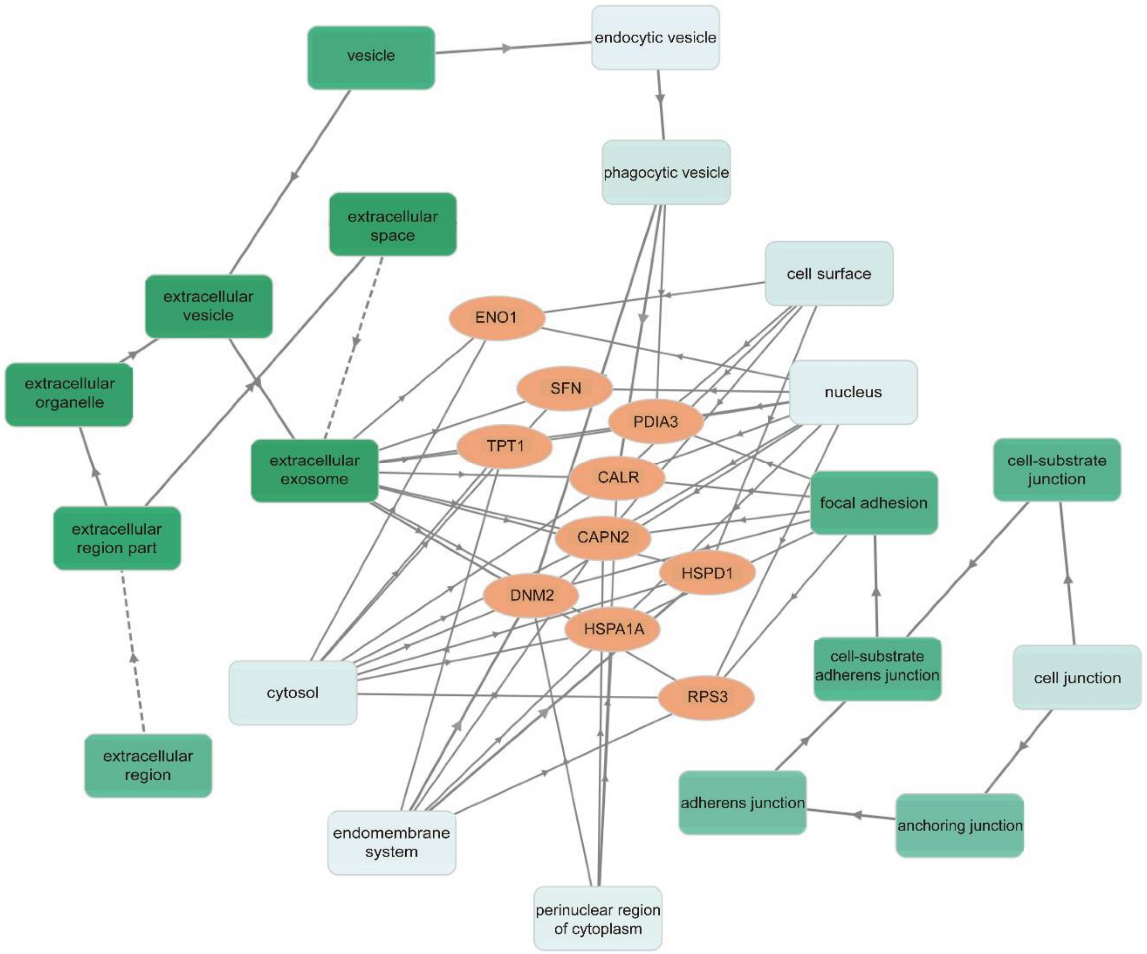

Supplementary Figure S1. Bioinformatic analysis of F3 and F3.TSG (A) One-way hierarchical clusting heat map; (B) Gene ontology (GO) biologicial process analysis; (C) GO cellular component analysis; (D) GO molecular function analysis; (E) Network map of F3 and F3.TSG cells.
